# Immunopathogenesis and Cytokine Pathways in Reactive Infectious Mucocutaneous Eruption (RIME) in Pediatric Population: Infectious Triggers and Molecular Insights

**DOI:** 10.1007/s12016-026-09177-z

**Published:** 2026-06-25

**Authors:** Filippos Filippatos, Konstantinos Kakleas, Athanasios Michos

**Affiliations:** First Department of Pediatrics, Medical School, National and Kapodistrian University of Athens, ’Aghia Sophia’ Children’s Hospital, Athens, 11527 Greece

**Keywords:** Reactive infectious mucocutaneous eruption, *Mycoplasma pneumoniae*, Pediatric mucositis, Hyperinflammation, Proteomics, Cytokines

## Abstract

Reactive infectious mucocutaneous eruption (RIME) is increasingly recognized as a pediatric syndrome in which severe mucositis follows a recent infection and cutaneous involvement is usually limited. Although Mycoplasma pneumoniae remains the prototype trigger, influenza, SARS-CoV-2, adenovirus, respiratory syncytial virus, and other respiratory pathogens can produce a similar clinical phenotype. This narrative review summarizes pediatric RIME/MIRM literature and selected adult or comparator-disease evidence when direct pediatric data are unavailable, explicitly distinguishing direct RIME evidence from extrapolated mechanisms. This review synthesizes current evidence on the immunopathogenesis of RIME in children, with emphasis on how pathogen-specific factors intersect with innate and adaptive immune pathways to drive mucosal injury. We discuss the central roles of epithelial sensing, inflammasome activation, cytokine amplification, neutrophil recruitment, and T-cell polarization, and we relate these mechanisms to the severe oral, ocular, and anogenital disease that often defines pediatric presentations. We also integrate emerging proteomic data showing recurring molecular programs, including acute-phase and complement activation, neutrophil-associated epithelial injury, and interferon-inducible antiviral responses. In addition, we review the possible contribution of genetic susceptibility, especially variation in innate immune sensors and cytokine signaling pathways, to disease severity and recurrence. Finally, we address the main diagnostic challenges, histopathologic and imaging findings, differential diagnosis from Stevens-Johnson syndrome, erythema multiforme, and Kawasaki disease, and the implications of these insights for risk stratification and treatment. Taken together, current evidence supports RIME as a pathogen-triggered, mucosa-predominant inflammatory syndrome in which exaggerated host responses, rather than pathogen presence alone, shape the clinical phenotype.

## Introduction

 Reactive infectious mucocutaneous eruption (RIME) describes a spectrum of acute, infection-associated mucocutaneous inflammatory reactions in which mucosal disease is prominent and skin involvement is typically limited [1, 2]. In children, the syndrome can range from painful but self-limited mucosal erosions to severe multisite mucositis requiring hospitalization for hydration, pain control, ophthalmologic monitoring, or nutritional support [1, 2].

The clinical importance of RIME lies not only in the acuity of presentation but also in the diagnostic uncertainty that often surrounds it. The syndrome overlaps clinically with Stevens-Johnson syndrome/toxic epidermal necrolysis (SJS/TEN), erythema multiforme, and other inflammatory mucocutaneous disorders, yet its biology appears distinct [1–11]. Rather than reflecting a classic drug-triggered cytotoxic reaction, RIME is most often linked to a recent infection, particularly of the respiratory tract, and appears to arise from an exaggerated mucosal immune response to that trigger [1–4, 12–22].

Epidemiologic patterns also support an infectious framework. Pediatric cases tend to cluster during periods of increased circulation of respiratory pathogens, and the syndrome is reported most often in school-aged children and adolescents, the age groups in which common triggers such as *Mycoplasma pneumoniae*, influenza, adenovirus, RSV, and SARS-CoV-2 are frequent [1–3, 12–15, 17–19, 23–28]. At the same time, the clinical phenotype is not explained by pathogen exposure alone. Why one child develops transient respiratory symptoms while another develops severe oral, ocular, or genital mucositis likely depends on host response, including innate immune activation, cytokine balance, epithelial vulnerability, and perhaps genetic predisposition [29–43].

## Methods, Literature Search and Evidence Selection

This article is a narrative review rather than a systematic review. PubMed/MEDLINE and Google Scholar were searched through 31 May 2026 using combinations of the terms “reactive infectious mucocutaneous eruption”, “RIME”, “Mycoplasma-induced rash and mucositis”, “MIRM”, “pediatric mucositis”, “Stevens-Johnson syndrome”, “erythema multiforme”, “Kawasaki disease”, “MIS-C”, “ocular”, “treatment”, “proteomics”, “cytokines”, “inflammasome”, “interferon”, and names of candidate infectious triggers. Reference lists of key reviews and case series were manually screened.

Studies were prioritized in the following order: (1) pediatric RIME/MIRM systematic reviews, retrospective cohorts, case series, recurrent-disease cohorts, outbreak reports, and ocular reviews; (2) adult RIME reports only when they clarified terminology, recurrence, ocular disease, or management; (3) mechanistic studies directly performed in RIME/MIRM; (4) mechanistic studies from related mucocutaneous inflammatory disorders; and (5) pathogen-immunology studies used to support biologic plausibility. No formal risk-of-bias tool or meta-analysis was applied. Adult and non-RIME mechanistic data are identified as indirect evidence and were not used to make definitive claims about pediatric RIME.

## Terminology, Diagnostic Boundaries, and Strength of Evidence

The boundaries among RIME, MIRM, EM major, and SJS/TEN remain unsettled. MIRM is best reserved for the M. pneumoniae-associated phenotype with prominent mucositis and sparse skin disease, whereas RIME is broader and includes similar infection-associated mucocutaneous eruptions caused by other pathogens [1–5]. The added value of the present review is its pediatric immunopathogenesis focus: it links clinical phenotype to epithelial sensing, cytokine amplification, mucosal barrier injury, and the still-limited direct molecular evidence available in children. This broader term is useful clinically, but it should not imply that all infectious mucocutaneous eruptions share the same molecular mechanism.

Two practical boundaries are worth emphasizing. First, drug exposure does not automatically exclude RIME, because many children receive antipyretics or antibiotics after the infectious prodrome that ultimately triggers RIME. However, exposure to a high-risk drug in a plausible latency window, progressive dusky macules, extensive epidermal detachment, positive Nikolsky sign, or biopsy consistent with full-thickness epidermal necrosis should shift concern toward SJS/TEN and prompt immediate withdrawal of suspected medications Second, RIME can overlap clinically with KD and MIS-C through fever, conjunctival injection, mucosal changes, rash, and inflammatory markers, but cardiac involvement, shock, gastrointestinal disease, and sustained systemic hyperinflammation favor KD or MIS-C over isolated RIME [10, 12–14].

The evidence base is strongest for *M. pneumoniae*, moderate for SARS-CoV-2, influenza, adenovirus, and *Chlamydia pneumoniae* because of case reports, small series, and outbreak observations, and weaker for less common viral or enteric triggers where attribution often rests on single reports or coinfection contexts [18–29]. Therefore, trigger-specific mechanisms should not be presented as equivalent in certainty [1, 2, 4, 5, 10, 11].

This review examines the innate and adaptive pathways that underlie pediatric RIME, the pathogen-specific mechanisms that may shape different clinical patterns, and the emerging proteomic and genetic data that help explain severity, recurrence, and heterogeneity. By placing clinical features alongside molecular insights, we aim to provide a framework that is useful both diagnostically and therapeutically. Evidence hierarchy used in this narrative review is presented in Table 1.

**Table 1:** Evidence hierarchy used in this narrative review

Evidence category	Examples used in this review	Interpretation
Direct RIME/MIRM evidence	Foundational MIRM/RIME reviews, pediatric series, recurrence/outbreak data, ocular systematic review, treatment reports, and the recent small RIME immune-profiling study [1–3, 5, 6, 28, 30–44]	Supports clinical phenotype, recurrence, ocular risk, treatment experience, and limited direct immunologic observations.
Related mucocutaneous inflammatory disorders	SJS/TEN, EM, KD, MIS-C, and pediatric severe cutaneous adverse reaction reclassification studies [7–17, 45–47]	Used for differential diagnosis and mechanistic comparison, not as proof of RIME-specific biology.
Broader infection and mucosal immunology	Innate RNA/DNA sensing, TLR signaling, inflammasomes, interferon biology, and pathogen-specific immune studies [48–84]	Used only to build plausible models when direct pediatric RIME data are unavailable.

## Epidemiology

RIME remains underrecognized, and its true population incidence in children is still unknown. Most available data come from retrospective series, case reports, and cohorts centered on MIRM, which likely means that the overall burden of disease is underestimated and heavily influenced by local awareness, referral patterns, and access to respiratory pathogen testing [1–4, 15, 44–46].

Even with these limitations, some epidemiologic features are consistent. RIME occurs most often in school-aged children and adolescents, broadly mirroring the age distribution of common respiratory triggers such as *M. pneumoniae*, influenza, RSV, and adenovirus [2, 15, 18]. Cases in infants and adults do occur, but they are less common. No stable sex predominance has been established across pediatric series, although some reports of MIRM suggest a male excess; whether this reflects biology or referral bias remains uncertain [1–3, 15, 18].

Seasonality is another recurring feature. Pediatric cases often cluster during the winter respiratory season, when influenza, RSV, adenovirus, and *M. pneumoniae* circulate more widely [2, 17, 19, 26]. During the COVID-19 pandemic, periods of high SARS-CoV-2 transmission were accompanied by increasing recognition of SARS-CoV-2-associated RIME, which broadened the accepted trigger spectrum and reinforced the parainfectious model [6, 12–14, 16, 20–22]. Geographic differences in reported triggers likely reflect underlying regional pathogen epidemiology as well as differences in testing practices and specialist access [2].

Outbreak patterns are also informative. Clusters of RIME or MIRM have been described in the setting of school-based or community respiratory outbreaks, consistent with the idea that the syndrome represents a relatively uncommon host response to otherwise common infections [2, 12–14, 17, 18, 25, 47]. Most affected children are previously healthy, although RIME is not confined to that group, and current evidence is insufficient to determine whether immunocompromise independently increases risk [2, 15, 17, 44].

Pediatric case series and outbreak observations should anchor clinical interpretation more strongly than mechanistic extrapolation. Available pediatric MIRM/RIME cohorts and recurrent-disease series provide the most direct evidence for the clinical phenotype and should therefore be given priority when interpreting disease presentation, severity, recurrence, and treatment needs. Across these studies, pediatric RIME is consistently characterized by prominent oral mucositis, often with hemorrhagic crusting, erosions, odynophagia, reduced oral intake, and the need for hospitalization primarily for hydration, analgesia, nutritional support, and mucosal care. Ocular and genital involvement are also frequent and clinically important, supporting the concept that RIME is a multisite mucosal syndrome rather than a primarily cutaneous eruption. In contrast, skin involvement is usually limited, sparse, targetoid, vesiculobullous, or erosive, and extensive epidermal detachment is uncommon, which helps distinguish RIME/MIRM from classic SJS/TEN in most pediatric presentations [15, 17, 18, 48].

Recurrent-disease series further suggest that RIME is not always a one-time reaction to a single pathogen. Recurrences may occur after repeated Mycoplasma pneumoniae infection or after exposure to different respiratory pathogens, supporting the possibility that a subset of children has a durable host-response susceptibility to mucosal hyperinflammation. Importantly, these observations come from clinical cohorts rather than from extrapolated cytokine or genetic models, and they therefore provide a more reliable basis for practical recommendations on follow-up, counseling, trigger testing, and early recognition of relapse. The 2024 Sydney outbreak of M. pneumoniae-associated RIME also illustrates the epidemiologic link between community respiratory pathogen surges and clustered mucocutaneous presentations, reinforcing the view that RIME may emerge as a recognizable complication during periods of increased pathogen circulation [15, 17, 18, 48].

These pediatric clinical data should therefore function as the foundation of the review’s interpretive framework. Mechanistic hypotheses involving epithelial sensing, inflammasome activation, complement amplification, neutrophil recruitment, interferon-inducible pathways, and cytokine-defined endotypes are useful for explaining why this phenotype may occur, but they should be interpreted in light of what pediatric cohorts actually show: mucosa-predominant disease, frequent oral–ocular–genital involvement, hospitalization driven mainly by supportive-care needs, generally favorable recovery, and occasional recurrence. In this way, case series and outbreak observations help prevent overextension of mechanisms borrowed from MIS-C, SJS/TEN, severe COVID-19, or general respiratory infection immunology, while still allowing those mechanisms to be discussed as plausible, hypothesis-generating explanations for the observed clinical pattern [15, 17, 18, 48].

Recurrence appears to occur in a minority of patients. When it does, it is often temporally linked to reinfection or to exposure to a different respiratory pathogen, suggesting that at least some children carry an enduring inflammatory susceptibility rather than a response limited to a single organism [2, 8, 60, 68, 69]. At present, however, no standardized preventive strategy exists beyond routine infection control and careful follow-up after severe or recurrent disease. Adult recurrent RIME reports are useful for demonstrating trigger heterogeneity across episodes but should not be assumed to define pediatric risk without pediatric confirmation.

## Diagnostic and Clinical Assessment

RIME is best understood clinically as a mucosa-predominant syndrome characterized by severe mucositis, usually involving at least two mucosal sites among the oral, ocular, and anogenital surfaces, in temporal association with a recent infection and without the drug exposure pattern typical of SJS/TEN [1–4, 15]. In current pediatric practice, the term encompasses classic *Mycoplasma pneumoniae*-induced rash and mucositis (MIRM) as well as comparable postinfectious phenotypes associated with respiratory viruses such as influenza, adenovirus, RSV, and SARS-CoV-2, and with selected bacterial triggers including group A *Streptococcus* [1–4, 12–15, 19–22, 25, 47, 48]. Recent case-based literature also reinforces that RIME can occur with sparse or absent skin disease and with a broad infectious trigger spectrum [29].

The diagnosis remains primarily clinical. A careful history should establish the sequence of events, especially whether respiratory, constitutional, or gastrointestinal symptoms preceded the eruption by days to weeks. Recent school or household outbreaks, cough, fever, sore throat, conjunctivitis, and medication exposure all matter, particularly because a recent high-risk drug exposure would shift concern toward SJS/TEN rather than RIME [1–4, 15, 45, 47, 49, 50]. On examination, the most striking feature is usually severe mucositis rather than extensive skin disease. Oral involvement may include diffuse stomatitis, hemorrhagic crusting, erosions, and marked odynophagia. Ocular disease ranges from conjunctivitis and discharge to photophobia, pseudomembranes, or early symblepharon, while genital disease may present with painful erosions, dysuria, or vulvovaginitis. Skin lesions, when present, are often sparse, targetoid, or vesiculo-erosive, with much less epidermal detachment than would be expected in SJS/TEN [1–5, 45, 47, 50]. Drug exposure should therefore be interpreted by timing and phenotype rather than treated as an absolute exclusion: antibiotics, antipyretics, or analgesics may be started after the infectious prodrome, whereas exposure to a high-risk medication within a compatible latency window, progressive skin pain, dusky macules, or epidermal detachment should prompt urgent SJS/TEN assessment, culprit-drug withdrawal, and reconsideration of RIME only if the eruption remains mucosa-predominant and infection-linked [1–9].

Initial assessment should also identify red flags that justify admission and multidisciplinary care. These include inability to maintain hydration because of oral pain, inability to handle secretions, significant ocular pain or photophobia, urinary retention, severe genital pain, or uncontrolled pain requiring escalation beyond routine supportive care. In many children, the burden of disease is driven less by systemic instability than by mucosal pain, nutritional compromise, and the risk of ocular sequelae [2, 4, 45, 47, 49, 50].

Ocular assessment should be structured from the time of presentation because ocular involvement may progress rapidly and may be underestimated when cutaneous disease is limited. In children with RIME/MIRM, ocular disease can range from mild conjunctival injection to severe inflammatory ocular-surface disease with epithelial defects, pseudomembranes, membrane formation, corneal involvement, and early cicatricial change. Therefore, the initial examination should specifically document ocular pain, photophobia, tearing, discharge, reduced visual acuity, conjunctival hyperemia, eyelid margin involvement, corneal staining, pseudomembranes, membrane formation, and any early adhesions or symblepharon. The presence of ocular pain, photophobia, reduced visual acuity, corneal staining, pseudomembranes, membrane formation, or early symblepharon should prompt urgent ophthalmology review rather than delayed outpatient assessment [45, 47, 50].

This approach is important because ocular disease in RIME may evolve quickly, even when skin involvement is sparse and the overall phenotype appears less severe than SJS/TEN. Early ophthalmologic involvement allows prompt institution of ocular-surface protection, including frequent preservative-free lubrication, careful removal or management of membranes when indicated, topical anti-inflammatory or antimicrobial therapy directed by ophthalmology, and monitoring for corneal epithelial injury. These measures are not merely symptomatic; they are intended to reduce the risk of late complications such as conjunctival scarring, symblepharon, dry eye disease, keratitis, limbal damage, and persistent visual morbidity [45, 47, 50].

Ocular findings may also assist the differential diagnosis. RIME often presents with prominent mucositis and conjunctival inflammation but limited epidermal detachment, whereas SJS/TEN more commonly produces widespread epidermal necrosis with high risk of severe ocular-surface injury. Kawasaki disease and MIS-C may include conjunctival injection, but this is typically non-purulent and occurs in the context of systemic vasculitic or multisystem inflammation rather than erosive mucositis. For this reason, ocular assessment in suspected RIME should not be limited to confirming conjunctivitis; it should actively evaluate severity, progression, and features that would change diagnosis, treatment intensity, or follow-up planning. Children with significant ocular involvement should receive follow-up after discharge, because cicatricial complications and dry eye symptoms may become clinically apparent after the acute mucocutaneous eruption has improved [45, 47, 50].

Laboratory studies are useful mainly for documenting systemic inflammation, identifying the infectious trigger, and recognizing hyperinflammatory complications. Common findings include leukocytosis with neutrophilia and elevated CRP, ESR, and ferritin. In more severe cases, cytokine profiling may show increased IL-6, TNF-α, IL-1β, or IFN-γ, supporting the presence of a hyperinflammatory phenotype, although such testing is not universally available [15, 29, 34–37, 48]. Testing directed at specific pathogens, including PCR and serology for *M. pneumoniae*, influenza, SARS-CoV-2, adenovirus, and other suspected triggers, can help support the diagnosis and may inform treatment.

Biopsy is not mandatory in typical cases but can be helpful when the differential diagnosis is broad or when drug-induced epidermal necrolysis remains a concern. Histopathology in pediatric RIME often shows epidermal necrosis with a denser inflammatory infiltrate than is typically seen in SJS/TEN, including lymphocytes, neutrophils, and sometimes subepidermal blistering. By contrast, full-thickness epidermal necrosis with sparse inflammation and widespread keratinocyte apoptosis should raise greater concern for SJS/TEN [5–9, 51–53]. In selected severe cases, immunohistochemistry may demonstrate activated T cells and macrophages together with increased IL-6, TNF-α, and IFN-γ expression, further supporting an inflammatory, parainfectious process rather than a purely cytotoxic drug reaction.

## Treatment Strategies in Pediatric RIME

Supportive care remains the foundation of treatment in pediatric RIME and should be initiated early, because the immediate morbidity is usually driven by mucosal pain, dehydration, impaired oral intake, ocular-surface inflammation, and genitourinary discomfort rather than by extensive epidermal loss. Children should be admitted when oral pain prevents adequate hydration or nutrition, when secretions cannot be handled safely, when pain control requires escalation, or when ocular, genital, urinary, or airway involvement is clinically significant. Core supportive measures include aggressive analgesia, topical oral care, hydration, electrolyte monitoring, nutritional support, secretion control, wound and mucosal care, and prevention or treatment of secondary infection. Early multidisciplinary involvement is particularly important in moderate to severe cases: ophthalmology should be involved when conjunctivitis is painful, photophobia is present, corneal staining is suspected, membranes or pseudomembranes are seen, or visual symptoms occur. Dermatology can help distinguish RIME from SJS/TEN, erythema multiforme, and autoimmune blistering disease, infectious diseases input can guide pathogen testing and antimicrobial therapy, while gynecology or urology may be needed for vulvovaginal, penile, urethral, dysuric, or urinary-retention symptoms [4, 44, 45, 47, 50].

Pathogen-directed therapy should be used when a treatable infectious trigger is suspected or confirmed, but it should not be viewed as sufficient therapy for established immune-mediated mucositis. In suspected or confirmed Mycoplasma pneumoniae-associated RIME/MIRM, macrolide therapy is commonly used, both to treat the underlying infection and to reduce ongoing pathogen-driven immune stimulation. Testing should be interpreted carefully, because PCR may reflect acute infection while serology may require paired samples or clinical correlation. Antiviral or antibacterial treatment should similarly be considered for other treatable triggers when supported by microbiological testing, epidemiologic context, or clinical severity. However, once the mucocutaneous eruption is established, tissue injury is likely sustained by host inflammatory pathways, including epithelial danger signaling, cytokine amplification, neutrophil recruitment, and possibly humoral or complement-mediated mechanisms. Therefore, antimicrobial therapy alone may not rapidly reverse oral, ocular, or anogenital inflammation, and supportive care remains essential even when an organism-directed treatment is started [1–4, 15, 44, 51–59].

Systemic corticosteroids are frequently used in moderate to severe RIME, particularly when mucositis compromises hydration or nutrition, when ocular or genital disease is significant, or when inflammation is progressive despite optimized supportive care. The rationale is to interrupt cytokine-driven mucosal inflammation, reduce edema and pain, and possibly to shorten the period of functional impairment. In practice, corticosteroids are often considered when oral intake is poor, hospitalization is required, ocular inflammation is more than mild conjunctival injection, genital erosions are painful or obstructive, or systemic inflammatory markers are high. Nevertheless, the evidence base remains limited, consisting mainly of retrospective cohorts, case series, and case reports rather than randomized pediatric trials. As a result, dose, route, timing, duration, and tapering strategy are not standardized, and treatment decisions should be individualized according to disease severity, diagnostic certainty, infection status, and multidisciplinary assessment [4, 44, 48, 60].

IVIG has also been used in severe RIME or in cases where the diagnostic boundary with Kawasaki disease, MIS-C, or SJS/TEN remains uncertain, but its role in RIME is not established. In some children, IVIG may be chosen because the presentation includes persistent fever, marked systemic inflammation, conjunctival involvement, mucosal disease, and diagnostic overlap with other pediatric hyperinflammatory syndromes. However, unlike Kawasaki disease and MIS-C, where IVIG is embedded in disease-specific treatment pathways to reduce systemic and cardiac complications, RIME does not currently have a validated IVIG-based treatment algorithm. Published experience is heterogeneous, and comparative data are insufficient to determine whether IVIG improves recovery beyond supportive care, antimicrobials, and corticosteroids in typical RIME. Thus, IVIG should be framed as an option for selected severe or diagnostically ambiguous cases rather than as routine first-line therapy [4, 44, 48, 60].

For severe, progressive, recurrent, or steroid-refractory RIME, targeted immunomodulation has been reported, but these approaches should be regarded as individualized rescue strategies rather than standard treatment. Cyclosporine has been used in severe mucocutaneous immune-mediated disease because of its ability to suppress T-cell activation, while TNF-α blockade with etanercept has been reported in small RIME/MIRM experiences and is biologically plausible when TNF-driven epithelial injury is suspected. Recent RIME-specific immune profiling showing TNF and interferon pathway dysregulation provides a mechanistic rationale for considering selected targeted therapy in exceptional cases. However, these data do not yet establish validated cytokine-defined endotypes that can reliably guide treatment selection, and the available clinical evidence remains limited. Decisions to use cyclosporine, etanercept, IL-1 blockade, IL-6 blockade, or other biologic strategies should therefore occur in multidisciplinary settings, with careful reassessment for persistent infection, evolving SJS/TEN, MIS-C, Kawasaki disease, autoimmune blistering disease, or other alternative diagnoses [29, 44, 61, 62].

This therapeutic framework differs from the management of Kawasaki disease, MIS-C, and SJS/TEN, even though these disorders may overlap clinically with RIME at presentation. In Kawasaki disease, treatment is directed toward systemic vasculitis and prevention of coronary artery aneurysms, with IVIG and aspirin forming the core of standard therapy and cardiology follow-up being essential. In MIS-C, treatment is guided by the degree of systemic inflammation, shock, myocardial dysfunction, and organ involvement, commonly using IVIG, corticosteroids, and selected biologic agents according to severity. In SJS/TEN, by contrast, immediate withdrawal of the culprit drug, supportive care in an appropriate high-acuity or burn-unit-level environment, fluid and wound management, infection prevention, ophthalmologic care, and necrolysis-directed therapy dominate early management. RIME differs from each of these conditions because treatment is centered on mucosal supportive care, identification and treatment of an infectious trigger when possible, and selective immunomodulation only when disease is severe, progressive, recurrent, or refractory [5, 8, 9, 63–66].

Treatment of pediatric RIME should follow a stepwise and severity-based approach. Mild cases may recover with analgesia, oral care, hydration, and close follow-up. Moderate cases often require hospitalization for pain control, fluids, nutrition, and specialist assessment, with pathogen-directed therapy when indicated. Severe cases with ocular-surface disease, genital complications, inability to maintain hydration, marked systemic inflammation, or diagnostic uncertainty may require systemic corticosteroids and, in selected circumstances, IVIG or targeted immunomodulation. Because current evidence remains largely observational, treatment should be described as pragmatic and individualized rather than protocolized, and future studies should define standardized severity criteria, treatment thresholds, outcome measures, and comparative effectiveness of corticosteroids, IVIG, cyclosporine, and biologics in pediatric RIME [4, 44, 45, 47, 48, 50, 60–62].

## Pediatric Immunopathogenesis of RIME

In the following sections, the strength of evidence is interpreted using a three-tier framework. The first tier includes findings reported directly in patients with RIME or MIRM, such as clinical phenotype, trigger attribution, mucosal distribution, recurrence patterns, selected histopathologic observations, and the limited available immune-profiling data from biologically characterized cases. These data provide the strongest basis for conclusions about pediatric RIME, but the number of direct mechanistic cohorts remains small, and most available studies are retrospective, case-based, or limited by heterogeneous pathogen testing and sampling time points [1, 2, 29, 34–37].

The second tier includes findings supported by related mucocutaneous or pediatric hyperinflammatory disorders, including Stevens–Johnson syndrome/toxic epidermal necrolysis, erythema multiforme, Kawasaki disease, and MIS-C. These include some clinical or immunologic features observed in RIME such as mucosal inflammation, epithelial injury, cytokine amplification, endothelial activation, complement involvement, or ocular complications. However, they differ substantially in triggers, tissue tropism, dominant immune mechanisms, prognosis, and treatment priorities. Therefore, evidence from these disorders is useful for contextualizing possible pathways in RIME but should not be presented as proof that the same mechanisms operate in RIME with the same intensity or clinical significance [1, 2, 29, 34–37].

The third tier includes hypothesis-generating mechanisms extrapolated from broader respiratory viral, atypical bacterial, and mucosal immunology. This category includes pathogen-sensing pathways such as TLR, RIG-I-like receptor, cGAS-STING, and inflammasome signaling; downstream IL-1, IL-6, TNF-α, interferon, CXCL8, and CXCL10 responses; neutrophil recruitment, epithelial barrier injury, complement amplification, and proposed genetic susceptibility pathways. These mechanisms are biologically plausible and help explain how different infectious triggers may converge on a mucosa-predominant inflammatory phenotype, but they have not been validated as RIME-specific pathways in most cases. They should therefore be interpreted as conceptual models and research priorities rather than established biomarkers, therapeutic endotypes, or diagnostic criteria [1, 2, 29, 34–37].

This distinction is essential because pediatric RIME currently has a much stronger clinical evidence base than mechanistic evidence base. Direct pediatric case series and outbreak observations support the syndrome as an infection-associated, mucosa-predominant eruption, whereas many proposed molecular pathways are inferred from related diseases or from general host responses to respiratory pathogens. Throughout this review, mechanistic conclusions should therefore be weighted according to the source of evidence: direct RIME/MIRM observations first, related mucocutaneous inflammatory disorders second, and broader infection-immunology extrapolation third. This hierarchy helps preserve the clinical usefulness of the review while avoiding overstatement of proteomic, cytokine, genetic, or endotype-based claims that remain preliminary in pediatric RIME.

At the center of pediatric RIME is a mucosal immune response that is both rapid and disproportionate. Epithelial cells and resident myeloid cells in the oral, ocular, and anogenital mucosa are well equipped to sense invading pathogens through pattern-recognition receptors, including Toll-like receptors, RIG-I-like receptors, and DNA-sensing pathways such as cGAS-STING [30–33, 42, 70–73]. Once activated, these pathways converge on NF-κB and interferon regulatory factors, resulting in the production of proinflammatory cytokines, type I interferons, chemokines, and adhesion molecules. In practical terms, this means that an infection which initially begins as a respiratory or systemic illness can quickly be translated into intense mucosal inflammation.

RNA sensing and interferon pathways are relevant to the proposed RIME framework, particularly for virally triggered cases, but should be interpreted with caution. Viral RNA can be detected by endosomal and cytosolic pattern-recognition receptors, including TLR3, TLR7/8, RIG-I, and MDA5 leading to activation of interferon regulatory factors and downstream type I and type III interferon responses [30–33]. These pathways induce interferon-stimulated genes and chemokines such as CXCL10, that may contribute to antiviral defense, epithelial activation, leukocyte recruitment and mucosal inflammation [30–33]. This model is especially plausible for RIME-like eruptions associated with influenza, SARS-CoV-2, RSV, rhinovirus, parainfluenza virus, human metapneumovirus, enteroviruses, and other RNA viruses [30–33]. However, these mechanisms are not yet proven to represent universal or disease-defining pathways in RIME. Much of the evidence for RIG-I-like receptor activation, type I interferon signaling, and interferon-inducible chemokine responses comes from broader antiviral immunology, respiratory viral infection studies, and related pediatric inflammatory syndromes rather than from large, prospectively characterized RIME cohorts [30–33]. Therefore, RNA-sensing and interferon pathways should be described as biologically plausible and particularly relevant to viral-triggered RIME, but not as validated biomarkers, diagnostic criteria, or established therapeutic endotypes for all RIME patients. This distinction is important because different triggers may converge on a similar mucosa-predominant phenotype through different upstream signals. In viral-triggered RIME, interferon-linked pathways may be prominent. In *Mycoplasma pneumoniae*-associated disease, TLR2/6-mediated NF-κB activation, IL-1/IL-6/TNF amplification, Th1/Th17 polarization, and humoral or complement-associated mechanisms may be more relevant [30–33]. Thus, interferon biology should be integrated into a broader, trigger-specific model of RIME rather than presented as a single unifying mechanism for the entire syndrome [30–33].

The epithelial compartment is especially important. Viral cytopathy, bacterial toxins, and local tissue stress all damage the mucosal barrier and release danger-associated molecular patterns, which amplify signaling through Toll-like and NOD-like receptors and can activate the inflammasome [30–33, 42, 43, 58, 59, 64–73]. Caspase-1-dependent maturation of IL-1β and IL-18 then intensifies inflammation further, promoting edema, pain, and epithelial breakdown. This helps explain why pediatric RIME is so often dominated by painful erosive mucositis rather than by widespread epidermal detachment.

The older pathogenic hypotheses should be considered as complementary and not obsolete. Earlier models of Mycoplasma pneumoniae-associated mucocutaneous disease emphasized humoral and antibody-mediated mechanisms, particularly B-cell activation, immune-complex deposition in mucocutaneous tissues, complement activation, and molecular mimicry between microbial antigens and epithelial or keratinocyte targets [1, 2, 4, 18, 54–57]. These mechanisms remain biologically plausible and may help explain several features of RIME/MIRM, including the delayed timing after respiratory infection, the mucosal predominance despite limited cutaneous detachment, and the occurrence of disease in only a minority of infected children [1, 2, 4, 18]. Newer models centered on epithelial pattern-recognition signaling, inflammasome activation, IL-1/IL-6 amplification, interferon-inducible pathways, and neutrophil-associated epithelial injury should therefore not be interpreted as replacing these older concepts. Instead, RIME is better understood as a layered host-response syndrome in which infection-triggered antibody responses, immune complexes, complement activation, epithelial danger signaling, and cytokine amplification may operate simultaneously or sequentially. In this integrated model, humoral and complement-mediated mechanisms may initiate or localize tissue injury, whereas innate cytokine pathways and recruited neutrophils may amplify mucosal inflammation and determine clinical severity. Direct mechanistic evidence in pediatric RIME remains limited, and these pathways should be presented as complementary, hypothesis-generating components of a unified pathophysiologic framework rather than as independently validated disease endotypes.

Chemokine and adhesion-molecule signaling shape where the inflammation goes. Upregulation of CXCL8, CXCL10, ICAM-1, and VCAM-1 facilitates the recruitment of neutrophils and activated lymphocytes from the circulation into the mucosa [29, 34–37, 74, 75]. The result is a dense, localized inflammatory infiltrate that can be clinically dramatic even when skin involvement is minimal. This pattern is especially relevant in children, who are repeatedly exposed to respiratory pathogens and often mount brisk mucosal innate responses.

Adaptive immunity adds a second layer of amplification. Th1- and Th17-skewed responses appear particularly relevant, with IFN-γ and IL-17 contributing to epithelial injury, leukocyte recruitment, and persistence of inflammation [18, 29, 54–57]. Regulatory T cells should, in theory, limit this process, but in severe presentations the balance may tilt toward unchecked inflammatory signaling. In this setting, hypercytokinemia becomes clinically visible: fever, malaise, marked mucosal swelling, and elevated inflammatory markers all reflect the systemic spillover of what begins as a mucosal immune event [29, 34–37]. Direct pediatric RIME data proving a consistent Th1/Th17 polarization pattern are limited; therefore, IL-17- and Th17-related language in this review should be read as a plausible model derived from *Mycoplasma pneumoniae* and mucosal inflammatory biology, not as a validated diagnostic signature for RIME [29, 34–43].

Children may be especially vulnerable to this pattern because their immune systems combine strong innate responsiveness with evolving adaptive regulation. In school-aged patients, recurrent exposure to common viral and atypical bacterial pathogens may repeatedly prime mucosal tissues, which could help explain why RIME peaks in these age groups and why cytokine signatures in pediatric disease often appear more pronounced than in adults with similar infectious triggers [15, 18, 29, 34–37].

## Pediatric Pathogen-Specific Immunopathogenesis

The evidence supporting different triggers is not equivalent. *Mycoplasma pneumoniae* remains the prototype and best-supported trigger, with systematic reviews, pediatric cohorts, and outbreak data. Influenza, SARS-CoV-2, adenovirus, RSV, group A Streptococcus, and other pathogens are increasingly reported but are supported more often by case reports, small series, or outbreak-associated observations. Therefore, they should be presented as an expanding trigger spectrum rather than as equally established causes [1–3, 12–15, 17–22, 25–28].

Although RIME converges on a recognizably similar clinical phenotype, different pathogens appear to reach that phenotype through somewhat different immune routes. These distinctions likely matter, because they help explain why some children present with a strongly antiviral, IFN-rich signature, while others show a more neutrophilic, IL-1/IL-6-dominant picture.

### *Mycoplasma pneumoniae*

*Mycoplasma pneumoniae* remains the most established trigger in pediatric RIME. Its lipoproteins strongly engage TLR2/TLR6 on epithelial and myeloid cells, activating MyD88-, NF-κB-, and MAPK-dependent signaling and driving rapid production of IL-1β, IL-6, TNF-α, and CXCL8 [54–59]. In parallel, ciliary dysfunction and CARDS toxin-mediated epithelial injury promote release of danger signals, with secondary activation of NLRP3 and maturation of IL-1β and IL-18. Antigen presentation can further skew the response toward Th1 and Th17 pathways, increasing IFN-γ and IL-17 and reinforcing neutrophil recruitment and endothelial activation through ICAM-1 and VCAM-1. Clinically, this combination maps well onto the dense, painful mucositis and relatively limited skin disease that typify MIRM and related RIME phenotypes [1–3, 15, 18, 48, 49, 54–59, 76–81].

### *Chlamydia pneumoniae*

*Chlamydia pneumoniae* appears to trigger a related but somewhat more persistent inflammatory response. Through TLR2, TLR4, and inflammasome-associated pathways, it promotes IL-1β, IL-18, CXCL8, and CXCL10 production while its intracellular persistence may prolong epithelial and endothelial activation [82–85]. Chlamydial heat-shock proteins may function as chronic danger signals, sustaining NF-κB activity and tissue injury beyond the initial infectious phase. In children, this may translate into more prolonged mucosal erosions and a tendency toward recurrence with later infections [82–85].

### Influenza Viruses

Influenza-associated RIME is shaped primarily by antiviral sensing. Endosomal TLR3 and cytosolic RIG-I recognize viral RNA and drive IRF3/7-dependent type I interferon responses, while NF-κB-dependent pathways generate IL-6, TNF-α, GM-CSF, and CXCL10 [19, 86–89]. Viral antagonists such as NS1 modulate the timing and intensity of that response, but when inflammation is robust, complement activation and endothelial signaling add a second layer of injury. In pediatric cases, this tends to produce a strong IFN-inducible proteomic signature together with acute-phase activation, correlating clinically with severe stomatitis, conjunctivitis, and marked systemic symptoms [19, 86–89].

### SARS-CoV-2

SARS-CoV-2 also produces a strong innate immune signal, but with a characteristic imbalance between antiviral interferon responses and NF-κB-driven cytokine production. Viral RNA activates TLR7/8 and RIG-I-like pathways, yet several viral proteins blunt effective early interferon signaling. The result can be a paradoxical pattern of reduced early antiviral control alongside high IL-6, IL-1β, TNF-α, and CXCL10 [6, 12–14, 16, 20–22, 90, 91]. Complement activation, endothelial dysfunction, and coagulation abnormalities may further amplify mucosal edema and injury. In children with SARS-CoV-2-associated RIME, the phenotype is often severe but transient and usually remains mucosa-predominant rather than progressing to a multisystem inflammatory syndrome, although overlap with MIS-C-like immune patterns may occasionally occur [6, 12–14, 16, 20–22, 90, 91].

SARS-CoV-2-associated RIME must be distinguished from MIS-C. Both can occur after SARS-CoV-2 exposure and can share fever, conjunctival injection, rash, oral changes, and elevated inflammatory markers, but MIS-C is typically multisystemic, often has gastrointestinal, myocardial, shock, or coronary involvement, and requires cardiac evaluation and protocolized immunomodulatory treatment. RIME is usually more mucosa-restricted, with limited epidermal detachment and without the sustained systemic vasculitic/cardiac phenotype expected in MIS-C [20–22, 63, 64].

### Herpesviruses and Related DNA Viruses

Herpesviruses engage innate immunity in somewhat different ways depending on the specific virus, but several recurring principles apply. HSV activates TLR2, TLR9, and cGAS-STING pathways and often drives strong IFN-γ responses together with cytotoxic T-cell activation, resulting in epithelial lysis and painful mucosal disease [92–96]. EBV adds a layer of immune modulation through latency proteins and viral IL-10-like signals, yet still promotes inflammatory injury around mucosal surfaces. CMV, by contrast, is more strongly associated with sustained IL-6, TNF-α, and IL-8 production and may prolong tissue inflammation through immune-evasion strategies that blunt effective viral clearance [92–96]. Varicella-zoster virus likewise activates TLR2-dependent inflammatory programs and Th1-skewed responses, contributing to cytokine-mediated mucocutaneous injury [93–95].

### Enteroviruses

Enteroviruses, including coxsackieviruses and enterovirus D68, are sensed mainly through TLR3 and MDA5 and therefore often generate strong type I interferon responses together with IL-6- and TNF-α-driven inflammation [97–100]. Epithelial cytolysis and pyroptosis-related pathways may intensify mucosal ulceration. In children, this often corresponds to a CXCL10-high antiviral signature, systemic malaise, and severe oral disease [97–100].

### Group A*Streptococcus*

Group A *Streptococcus* tends to generate a more abrupt, IL-1/IL-6/TNF-heavy inflammatory pattern. Cell wall components activate TLR2, while streptolysins and superantigens drive broad T-cell activation and additional cytokine release [48, 74, 74, 75]. Inflammasome signaling and neutrophil extracellular trap formation can further injure the epithelium. Although the organism has mechanisms to evade complement, acute-phase and complement amplification are still commonly detected systemically, consistent with the sudden, painful, erosive mucositis seen in some pediatric cases [48, 74, 74, 75].

### Adenovirus

Adenovirus is especially relevant in pediatric RIME because it can produce striking mucosal and ocular disease. Unmethylated viral DNA activates TLR9 and cytosolic DNA-sensing through cGAS-STING, resulting in strong type I interferon signaling together with IL-6 and IL-8 production [25, 47, 70–73, 101]. At the same time, epithelial cytopathic effects release DAMPs such as ATP and HMGB1, which may reinforce inflammasome activity. Clinically, adenovirus-associated RIME often features prominent conjunctival involvement, and its proteomic profile tends to combine antiviral signaling with acute-phase activation and marked neutrophil recruitment [25, 47, 70–73, 101].

### Rhinovirus, RSV, Parainfluenza, HMPV, and Human Bocavirus

These common respiratory viruses share overlapping epithelial antiviral pathways but differ in the balance between interferon signaling and inflammatory amplification. Rhinovirus activates TLR3 and MDA5, producing type I and type III interferons, CXCL10, IL-6, and IL-8, while epithelial alarmins such as IL-33 may further intensify local pain and edema [30–100, 102, 103]. RSV signals through TLR4 and RIG-I but also encodes proteins that blunt effective interferon responses, often shifting the phenotype toward persistent NF-κB activation, neutrophilic exudation, and barrier breakdown [23, 24, 26–28, 104]. Parainfluenza and human metapneumovirus similarly engage TLR3- and RIG-I-linked antiviral pathways while generating significant IL-6-, IL-8-, and TNF-α-mediated inflammation [105–111]. Human bocavirus appears to activate TLR2 and TLR3 pathways and is frequently encountered in coinfections, where it may contribute to a broader inflammatory burden rather than act as an isolated trigger [112–116]. In clinical terms, these viruses often produce IFN-rich RIME phenotypes, but RSV and hMPV tend to carry a more neutrophilic and exudative signature.

### Norovirus and Rotavirus

Enteric viruses can also trigger RIME, although less commonly than respiratory pathogens. Norovirus activates TLR7/8-associated antiviral and inflammatory pathways, while rotavirus is sensed mainly through TLR3 and RIG-I-like receptors [82, 117–122]. In both settings, type I interferons, IL-6, and TNF-α may rise substantially, and systemic inflammatory spillover may be sufficient to produce oral erosions and a RIME-like phenotype after an initial gastrointestinal illness [82, 117–122].

### Parvovirus B19 and Measles Virus

Parvovirus B19 is sensed through TLR9 and can provoke both interferon signaling and inflammasome-related inflammation, partly through the effects of the NS1 protein [86, 123–125]. Because of its erythroid tropism, it may also be accompanied by cytopenias, adding a systemic dimension to the clinical picture. Measles virus, in contrast, is notable for its capacity to suppress antiviral signaling and induce broader immune dysregulation. By impairing RIG-I-like receptor activation and reshaping dendritic cell function, measles can create a paradoxical state of immune suppression alongside NF-κB-biased cytokine production, complement activation, and severe mucocutaneous inflammation in susceptible hosts [87, 88, 126–130].

 Taken together, these trigger-specific pathways suggest that pediatric RIME is not a single molecular disease but a shared mucosal phenotype that emerges from overlapping combinations of innate sensing, cytokine amplification, epithelial injury, and immune modulation. Laboratory and cytokine markers used in pediatric RIME are summarized in Table 2, and pathogen-specific immunopathogenesis is summarized in Table 3.

**Table 2:** Laboratory and cytokine markers used in pediatric RIME

Assay	Typical finding in RIME	Clinical use	Notes	References
CBC	Leukocytosis with neutrophilia	Baseline/trajectory	Correlates with acute inflammation	[15, 29, 48]
CRP	Elevated	Severity assessment; monitoring	Acute-phase kinetics	[15, 29, 34–37, 48]
ESR	Elevated	Inflammation tracking	Slower than CRP	[15, 48]
Ferritin	Elevated	Gauge hyperinflammation	Consider MAS/HLH work-up if very high	[15, 29, 48]
IL-6	Often elevated	Severity stratification, target in severe disease (context-dependent)	Interpret with clinical picture	[29, 34–37]
TNF-α	Often elevated	Monitoring in advanced settings	Not universally obtained	[29, 34–37]
IL-1β	Sometimes elevated	Severity context	Suggests inflammasome activity	[29, 34–37]
IFN-γ	Variable elevation	Hyperinflammation context	Th1 activation marker	[29, 34–37]
Pathogen testing (PCR/serology)	Trigger identified (e.g., *M. pneumoniae*, influenza, SARS-CoV-2)	Etiologic confirmation, guides therapy	Paired serology if needed	[1–3, 12–17, 19–22, 25]

*CBC* complete blood count, *CRP* C-reactive protein, *ESR* erythrocyte sedimentation rate, *IFN* interferon, *IFN-γ* interferon-gamma, *IL* interleukin, *TNF-α* tumor necrosis factor-alpha, *PCR* polymerase chain reaction, *MAS* macrophage activation syndrome, *HLH* hemophagocytic lymphohistiocytosis

**Table 3:** Pathogen-specific immunopathogenesis in pediatric RIME

Pathogen/group	Primary PRRs	Key signaling	Dominant cytokines	Molecular and immune hallmarks	Clinical notes	References
*Mycoplasma pneumoniae*	TLR2/6	NF-κB, MAPK	IL-6, TNF-α, IFN-γ	↑ICAM-1/VCAM-1; Th1/Th17 skew	Common trigger, prominent mucositis	[1–3, 15, 18, 54–59]
*Chlamydia pneumoniae*	TLR2/4, inflammasome	NF-κB, Caspase-1	IL-1β, IL-18, CXCL8/CXCL10	Neutrophil-dominant chemokines	Chronic mucosal damage possible	[82–85]
Influenza viruses	RIG-I, TLR3	IRF3/7, NF-κB	Type I IFNs, IL-6, TNF-α	Antiviral/complement pathways ↑	Systemic symptoms prominent	[19, 86–89]
SARS-CoV-2	TLR7/8 (± RLRs)	NF-κB, dysregulated IFN	IL-6, IL-1β, TNF-α, impaired IFN	Acute-phase & complement ↑	Endothelial/coagulation effects	[6, 12–14, 16, 20–22, 90, 91]
Herpesviruses (HSV/EBV/CMV)	TLR2→NF-κB	NF-κB	IFN-γ (HSV), IL-10 (EBV), IL-6/TNF-α/IL-8 (CMV)	Inflammatory/apoptotic signaling ↑	Variable mucocutaneous disease	[92–96]
Enteroviruses (e.g., EV-D68)	TLR3; MDA5	IRF3/7, NF-κB	Type I IFNs, IL-6, TNF-α	IFN-responsive mediators ↑	Leukocyte recruitment	[97–100]
Group A Streptococcus	TLR2	NF-κB	IL-1β, IL-8, TNF-α	Acute-phase; complement ↑	Marked mucocutaneous damage	[48, 74, 74, 75]
Norovirus	TLR7/8	NF-κB, IRFs	IL-6, TNF-α, type I IFNs	Antiviral/inflammatory cascades ↑	Systemic inflammation in some	[117, 118]
Adenovirus	TLR9, cGAS–STING	IRF3/7, NF-κB	Type I IFNs, IL-6, IL-8	Antiviral/inflammatory mediators ↑	Pronounced mucosal inflammation	[25, 47, 70–73, 101]
Rhinovirus	TLR3, MDA5	IRF3/7, NF-κB	IL-6, IL-8, TNF-α	Inflammatory markers ↑	Upper airway predominant	[30–100, 102, 103]
Varicella-zoster virus	TLR2	NF-κB, Th1 skew	IL-6, IL-8, TNF-α, IFN-γ	Cytokine-mediated injury ↑	Can aggravate mucocutaneous disease	[92–95]
Parainfluenza virus	TLR3, RIG-I	IRF3/7, NF-κB	Type I IFNs, IL-6, IL-8, TNF-α	Cytokine/chemokine markers ↑	Severe mucosal inflammation	[105–108]
Respiratory syncytial virus	TLR4, RIG-I	IRF/NF-κB	IL-6, IL-8, TNF-α, type I IFNs	Inflammatory mediators ↑	Extensive mucosal damage possible	[23, 24, 26–28, 104]
Human metapneumovirus	RIG-I-like receptors	IRF/NF-κB	IL-6, TNF-α	Inflammatory markers ↑	Severe mucosal inflammation	[24, 109–111]
Human bocavirus	TLR2, TLR3	IRF/NF-κB	IL-6, TNF-α, IL-10	Inflammatory proteins ↑	Coinfections common	[112–116]
Rotavirus	TLR3; RLRs	IRF3/7, NF-κB	Type I IFNs, IL-6, TNF-α	Antiviral/inflammatory mediators ↑	GI mucosal involvement	[118–122]
Parvovirus B19	TLR9	IRF/NF-κB	Type I IFNs, IL-6, TNF-α, IFN-γ	Systemic inflammatory profile ↑	Erythropoiesis suppression	[86, 123–125]
Measles virus	Multiple (with evasion)	Impaired IFN, NF-κB	↑IL-6, IL-10, TNF-α, ↓effective IFN	Immune dysregulation proteome ↑	Extensive eruptions in severe cases	[87, 88, 126–130]

*PRR* pattern-recognition receptor, *PAMP* pathogen-associated molecular pattern, *TLR* Toll-like receptor, *RLR* RIG-I-like receptor, *RIG-I *retinoic acid-inducible gene I, *MDA5* melanoma differentiation-associated protein 5, *cGAS* cyclic GMP–AMP synthase, *STING* stimulator of interferon genes, *NF-κB* nuclear factor κB, *MAPK* mitogen-activated protein kinase, *IRF* interferon regulatory factor, *ICAM-1* intercellular adhesion molecule-1, *VCAM-1* vascular cell adhesion molecule-1, *RSV* respiratory syncytial virus, *hMPV* human metapneumovirus, *HBoV* human bocavirus, *VZV* varicella–zoster virus, *EBV* Epstein–Barr virus, *CMV* cytomegalovirus, *HSV* herpes simplex virus, *EV* enterovirus, *GAS* group A *Streptococcus*, *SARS-CoV-2* severe acute respiratory syndrome coronavirus 2

## Pediatric Proteomics in RIME

Direct RIME-specific molecular evidence remains limited and should not be overinterpreted as a validated proteomic signature. A recent RIME-specific study performed single-cell RNA sequencing in one adenovirus-triggered case and multiplex serum profiling in five active RIME patients, identifying TNF/IFN dysregulation, classical monocyte involvement, and increased CXCL9, CXCL10, IL-27, IL-6, and cytochrome c compared with healthy controls. Other acute-phase, complement, neutrophil, and antiviral modules discussed below are inferred partly from MIS-C, severe COVID-19, and pediatric respiratory viral cohorts rather than from large RIME cohorts [29, 34–37]. Endotypes based on IL-1, IL-6, CXCL10/IFN, neutrophil, or Th17 patterns should be considered hypothesis-generating. At present, RIME does not have validated cytokine-defined endotypes that can reliably guide risk stratification or treatment selection; any use of anti-IL-1, anti-IL-6, TNF-alpha blockade, or other biologics should be individualized for severe or refractory disease rather than biomarker-driven routine care [29, 34–37, 44, 61, 62].

Proteomic approaches have begun to clarify an important question in pediatric RIME: why children with similar triggers can look so different clinically. By examining proteins in serum, plasma, saliva, oral swabs, and tear fluid, these studies move beyond single cytokines and reveal broader inflammatory programs that better capture disease biology and severity [29, 34–37]. In practice, proteomics is useful because it helps connect bedside features, such as feeding intolerance, ocular inflammation, or disproportionate pain, with measurable molecular patterns. The term “proteomic” is used broadly here to include multiplex serum profiling and related molecular immune readouts; it should not imply that a reproducible, clinically validated RIME proteomic classifier already exists [29].

Across available pediatric datasets, several proteins recur consistently. Acute-phase reactants such as CRP and serum amyloid A are frequently elevated, as are cytokines and chemokines such as IL-6, TNF-α, IL-17, CXCL8, and CXCL10. Adhesion molecules including ICAM-1 and VCAM-1, together with complement components, further support the idea that endothelial activation and innate amplification are central to the syndrome rather than merely secondary phenomena [29, 34–37]. Key biomarkers associated with severity are listed in Table 4.

**Table 4:** Pediatric molecular and serum biomarkers associated with RIME severity

Marker	Class	Rationale/association	Clinical utility	References
CRP	Acute-phase protein	Correlates with systemic inflammation/severity	Diagnosis, monitoring	[29, 34–37]
Serum amyloid A (SAA)	Acute-phase protein	Tracks acute inflammation	Monitoring	[29, 34–37]
CXCL8 (IL-8)	Chemokine	Neutrophil recruitment; mucosal inflammation	Severity stratification (advanced settings)	[29, 34–37]
CXCL10 (IP-10)	Chemokine	Type I IFN-linked; antiviral response	Phenotyping	[29, 34–37]
IL-6	Cytokine	Hyperinflammatory driver	Therapeutic targeting/monitoring	[29, 34–37]
IL-17	Cytokine	Th17-mediated tissue injury	Endotyping	[29, 34–37]
TNF-α	Cytokine	Amplifies mucosal damage	Monitoring	[29, 34–37]
ICAM-1	Adhesion molecule	Leukocyte trafficking; endothelial activation	Severity context	[29, 34–37]
VCAM-1	Adhesion molecule	Leukocyte adhesion/migration	Severity context	[29, 34–37]
Complement components	Complement proteins	Amplified innate effector activity	Phenotyping	[29, 34–37]
IL-1β	Cytokine	Inflammasome-linked mucosal inflammation; reported variably and mainly inferred from mechanistic models	Hypothesis-generating endotyping; not a validated treatment-selection marker	[29, 34–37, 43]

*SAA* serum amyloid A, *CRP* C-reactive protein, *CXCL8 (IL-8)* C-X-C motif chemokine ligand 8, *CXCL10 (IP-10)* C-X-C motif chemokine ligand 10, *ICAM-1* intercellular adhesion molecule-1, *VCAM-1* vascular cell adhesion molecule-1, *IFN* interferon, *IL* interleukin, *TNF-α* tumor necrosis factor-alpha

Three interrelated inflammatory modules appear to recur most consistently in children. The first is an acute-phase/complement amplification program, characterized by increased fibrinogen chains and complement components across the C3-C5 axis and terminal pathway during acute illness. This pattern is more consistent with pattern-recognition-driven innate amplification than with classical immune-complex disease, and it likely contributes to the mucosal edema, tenderness, and inflammatory pain seen at presentation [29, 34–37]. The second is a neutrophil-dominant epithelial injury module. CXCL8-high, ICAM-1-high, and VCAM-1-high profiles align with dense, erosive mucositis and offer a plausible mechanistic explanation for the severe pain and feeding intolerance that so often define pediatric RIME [29, 34–37]. The third is an interferon-inducible antiviral module, in which CXCL10 and related mediators rise early and are most prominent in virally triggered disease. Importantly, this IFN-high signature often coexists with IL-6- and TNF-α-driven inflammation, underscoring that antiviral and hyperinflammatory pathways frequently overlap rather than occurring in isolation [29, 34–37].

Proteomic patterns also vary by trigger. Respiratory viruses such as influenza, SARS-CoV-2, RSV, hMPV, and parainfluenza tend to produce CXCL10-rich antiviral signatures layered on top of IL-6, TNF-α, and complement activation. RSV and hMPV often add a more obvious neutrophilic or exudative component. Adenovirus stands out for combining strong DNA-sensing and interferon-linked signals with intense ocular surface inflammation. By contrast, *M. pneumoniae* and group A *Streptococcus* more often generate IL-1-, IL-6-, and IL-17-skewed profiles with endothelial activation and a distinctly neutrophil-forward pattern, which fits their tendency to cause severe mucositis despite relatively limited skin disease [29, 34–37].

From a clinical standpoint, the literature now supports three pragmatic inflammatory endotypes. The first is an IL-1-dominant endotype, characterized by a neutrophil-rich proteome with increased IL-1β and IL-18, consistent with strong inflammasome activation. In severe, steroid-refractory disease, this pattern may justify early consideration of IL-1-directed therapy. The second is an IL-6-dominant endotype, in which elevated IL-6 is accompanied by acute-phase fibrinogen and complement activation. This profile is more often associated with systemic inflammatory symptoms and may support escalation toward IL-6-targeted treatment when conventional therapy is insufficient. The third is an IFN-dominant endotype, defined by a CXCL10-high antiviral signature, most often seen in virally triggered disease. In this setting, a focused antiviral work-up is especially relevant, and excessive immunosuppression should be used cautiously, as improvement often parallels resolution of the underlying infection [29, 34–37].

For routine practice, a practical severity and response panel could combine ferritin and complete blood count with CRP, serum amyloid A, IL-6, CXCL8, CXCL10, ICAM-1, VCAM-1, and complement components such as C3 and C5. In hospitalized children with moderate to severe disease, sampling at presentation, at 48–72 h, and again around day 7 may capture peak inflammation and early response to therapy. Falling CRP, SAA, CXCL8, and CXCL10 would generally support recovery, whereas plateauing or rising profiles should prompt reassessment for persistent infection, progressive ocular disease, dehydration, or hyperinflammatory complications such as MAS/HLH [29, 34–37].

## Genetic Susceptibility and Biomarkers

No reproducible RIME-specific susceptibility loci or clinically useful genetic markers have been established. The genes and pathways discussed in this section are candidate mechanisms extrapolated mainly from severe COVID-19, severe RSV infection, inflammasome biology, and general innate immune signaling, and should be framed as future research directions rather than determinants of RIME severity or recurrence [38–43].

Current evidence suggests that pediatric RIME likely develops on a polygenic, infection-modulated background rather than from a single Mendelian defect. The most plausible candidate pathways are those that govern innate sensing of pathogens, inflammasome activation, and the magnitude of downstream IL-1, IL-6, TNF, and interferon signaling [38–43]. This makes biological sense: if RIME reflects an exaggerated mucosal inflammatory response to otherwise common infections, then inherited variation in those pathways could influence who develops severe disease, who recurs, and who recovers uneventfully.

The strongest mechanistic candidates are genes involved in TLR, RIG-I/MDA5, and cGAS-STING signaling, as well as inflammasome-related loci such as *NLRP3*. Variants in *IL6*, *IL6R*, *IL1B*, *IL1R*, *TNFA*, and related receptors may further influence the intensity and persistence of cytokine signaling [38–43]. Genetic variation in *TLR4* has been linked to severity in RSV disease, and impaired TLR7-mediated antiviral responses have emerged as clinically relevant in severe viral illness, particularly in boys and young men, making such pathways especially interesting in recurrent or unusually severe pediatric RIME [38–43].

One important point is what has not been found. Unlike SJS/TEN, where strong and reproducible HLA associations support a drug-specific cytotoxic mechanism, no comparable HLA signal has yet been established in RIME [5–9, 38–43]. This difference reinforces the idea that RIME is biologically distinct from drug-induced epidermal necrolysis and should not be forced into the same immunogenetic framework.

At present, these biomarkers remain more useful for mechanistic insight and future risk stratification than for routine clinical testing. Still, they offer a path toward more personalized management. Children with evidence of inflammasome-predominant disease may eventually be distinguished from those with IFN-dominant antiviral endotypes or IL-6-driven hyperinflammatory phenotypes. Candidate genes and their potential clinical relevance are summarized in Table 5.

**Table 5:** Candidate genetic susceptibility & biomarkers in pediatric RIME

Gene/locus	Pathway	Putative effect in pediatric RIME	Clinical implication	References
IL6/IL6R	Cytokine/receptor	Higher IL-6 signaling → hyperinflammation	Consider anti-IL-6 in severe cases	[38–43]
IL1B/IL1R	Cytokine/receptor	Inflammasome-driven IL-1β responses	Candidate for anti-IL-1 strategies	[38–43]
TNFA/TNFR	Cytokine/receptor	TNF-α–mediated tissue injury	Adjunctive immunomodulation context	[38–43]
TLR4	PRR (RSV, some bacteria)	Higher viral cytopathology-linked cytokinemia	Identify risk for severe mucositis with RSV-season triggers	[38–43]
TLR7	Endosomal PRR (ssRNA)	Impaired type I IFN; male-biased severity	Consider testing in severe/recurrent, esp. boys	[38–43]
DDX58 (RIG-I)/IFIH1 (MDA5)	RLRs (RNA)	Sets IFN/CXCL10 axis	Candidate for research; not for routine testing	[38–43]
MB21D1 (cGAS)/TMEM173 (STING)	DNA sensing	IFN-high endotype (adenovirus-rich)	Research use only	[38–43]
TLR8/TLR9	Endosomal PRRs	Viral RNA/DNA sensing; severe phenotypes in variants	Risk identification in severe cases	[38–43]
NLRP3	Inflammasome	IL-1β/IL-18 maturation; hyperinflammation	Anti-inflammasome strategies	[38–43]
IL1B/IL1R (± IL1RN)	Cytokine/receptor	Inflammasome-driven mucosal injury	IL-1 blockade candidate	[38–43]
IL6/IL6R	Cytokine/receptor	Acute-phase/complement amplification	Consider IL-6 blockade in selected severe cases	[38–43]
Other immune regulators	Regulatory	Set-points affecting cytokine cascades	Personalized therapy research	[38–43]

*TLR* Toll-like receptor, *NLRP3* NOD-, LRR- and pyrin domain-containing protein 3, *IL* interleukin, *TNF-α* tumor necrosis factor-alpha, *PRR* pattern-recognition receptor

## RIME and Other Comparable Conditions

Taxonomic boundaries remain unresolved. In practice, some cases sit between RIME, MIRM, erythema multiforme major, and infection-associated SJS/TEN-like disease; therefore, terminology should follow the dominant phenotype, trigger relationship, medication timing, extent of epidermal detachment, and histopathology rather than assume a fully settled classification [1, 2, 4, 5, 10, 11].

One of the main clinical challenges in pediatric RIME is distinguishing it from other mucocutaneous inflammatory disorders that share fever, mucosal involvement, and skin lesions but differ substantially in pathogenesis and management.

The most important distinction is from SJS/TEN. SJS/TEN is most often drug-induced and characterized by widespread epidermal necrosis, epidermal detachment, and severe multisite mucosal disease driven largely by cytotoxic T-cell responses and mediators such as granulysin [5–9, 53]. By contrast, RIME is infection-associated, typically presents with far less skin detachment, and appears to be driven more by mucosal innate immune amplification, cytokine cascades, and pathogen-triggered inflammation. This distinction is clinically critical because the diagnostic framing affects everything from medication review to prognosis and therapeutic escalation.

Erythema multiforme is another important mimic, but its phenotype is usually different. EM classically presents with targetoid lesions, often acrally distributed, and mucosal involvement is milder or absent in many cases. Immunologically, EM is more closely linked to delayed-type hypersensitivity, often in the setting of HSV, and does not usually show the same degree of cytokine-driven mucosal injury that defines RIME [10, 11].

Kawasaki disease can overlap with RIME through fever, conjunctival injection, oral mucosal changes, and elevated inflammatory markers, but the clinical context is different. Kawasaki disease affects younger children, is characterized by sustained systemic vasculitis, and carries a risk of coronary artery involvement that is not a feature of RIME [63, 65, 66]. Although both conditions may show elevated IL-1β, IL-6, and TNF-α, Kawasaki disease is fundamentally a systemic vasculitic syndrome, whereas RIME is better understood as a transient, infection-linked mucosal inflammatory eruption.

MIS-C is an additional pediatric differential diagnosis, particularly after SARS-CoV-2 exposure. Compared with RIME, MIS-C usually has more sustained systemic inflammation, gastrointestinal and cardiovascular involvement, myocardial dysfunction or shock in severe cases, and a requirement for cardiac evaluation and follow-up; RIME usually remains mucosa-predominant and lacks the coronary-artery surveillance pathway central to Kawasaki disease and MIS-C [63, 64].

Timing can assist but should not be used alone. RIME typically follows respiratory or systemic infection by several days to weeks; SJS/TEN most often appears within one to three weeks after starting a high-risk medication, or sooner with re-exposure; erythema multiforme often follows HSV or other infection; Kawasaki disease and MIS-C are defined more by persistent fever, systemic inflammation, and age/organ-pattern context than by isolated mucositis [1–11, 63–66].

Careful attention to the trigger, pattern of skin involvement, severity and distribution of mucositis, histopathology, and laboratory profile usually allows the distinction to be made. These differential features are summarized in Tables 6 and 7.

**Table 6:** Clinical and differential diagnosis summary (RIME vs. Stevens-Johnson syndrome/Toxin epidermal necrolysis vs. Erythema multiforme vs. Kawasaki disease)

Feature	RIME	Stevens-Johnson syndrome/Toxin epidermal necrolysis	Erythema multiforme	Kawasaki disease	References
Typical trigger(s)	Recent infection (e.g., *Mycoplasma pneumoniae*, influenza, SARS-CoV-2, others)	Usually medications (anticonvulsants, antibiotics); occasionally infection	Often HSV/other infections; post-infectious	Post-infectious immune activation; unknown exact trigger	[1–5, 12–15, 18–22, 25, 63–66]
Cutaneous lesions	Prominent mucositis, variable/limited skin lesions; erosions/ulcers	Widespread epidermal necrosis/detachment; dusky macules/bullae	Targetoid papules/plaques; acral predominance	Polymorphous rash; often truncal	[1–11, 15, 49]
Mucosal involvement	Severe (oral/ocular/genital) mucositis typical	Severe, multi-site	Usually mild/absent	Non-exudative conjunctivitis; strawberry tongue; fissured lips	[1–4, 15, 45, 47, 49, 50, 63, 65, 66]
Systemic signs	Fever, conjunctivitis, lymphadenopathy; often antecedent respiratory illness	High fever; potential multi-organ involvement	Mild fever or none	Prolonged fever; lymphadenitis; risk of coronary artery changes	[1–4, 15, 44, 63, 65, 66]
Key laboratory patterns	↑WBC/neutrophils; ↑CRP/ESR; ↑ferritin; cytokines variably ↑ (IL-6, TNF-α, IL-1β, IFN-γ)	Nonspecific inflammatory markers; ± transaminitis	Often nonspecific/normal	↑CRP/ESR; thrombocytosis (subacute); sterile pyuria	[15, 29, 34–37, 48, 63–66]
Histopathology (skin/mucosa)	Epidermal necrosis; dense lymphocytic/neutrophilic infiltrate; ± subepidermal blistering	Full-thickness epidermal necrosis; sparse dermal infiltrate; keratinocyte apoptosis	Interface dermatitis with necrotic keratinocytes; perivascular lymphocytes	Medium-vessel vasculitis; perivascular inflammation	[5–11, 51–53]
Course/prognosis	Parainfectious; may be severe but often self-limited; recurrences possible	Potentially life-threatening; high morbidity	Self-limited; recurrences common	Risk of coronary artery aneurysms without timely IVIG	[1–4, 15, 53, 60, 68, 69]
Management emphasis	Supportive care; treat trigger; consider immunomodulation in severe cases	Stop culprit drug; burns-unit care; immunomodulation as indicated	Supportive care; trigger management	IVIG + aspirin; cardiology follow-up	[4, 5, 8, 9, 44, 61–66]

**Table 7:** Comparison of RIME and MIS-C in children

Feature	RIME	MIS-C	Clinical implication	References
Typical setting	Days to weeks after respiratory/systemic infection; mucosa-predominant	Usually post-SARS-CoV-2 hyperinflammatory syndrome with multisystem involvement	SARS-CoV-2 exposure alone does not equal MIS-C; organ pattern matters	[63, 64]
Mucosal/skin pattern	Severe oral/ocular/genital mucositis with limited skin detachment	Conjunctivitis, oral changes, polymorphous rash may occur but mucositis is not usually the isolated dominant problem	Dominant mucositis with sparse skin favors RIME	[1–3, 63, 64]
Systemic involvement	Often inflammatory but usually not shock/cardiac-dominant	May include shock, myocarditis, ventricular dysfunction, gastrointestinal disease, coagulopathy	Obtain cardiac markers/echocardiography when MIS-C is plausible	[63, 64]
Treatment emphasis	Supportive care, trigger treatment, selective immunomodulation in severe disease	Protocolized immunomodulation, commonly IVIG and glucocorticoids, with cardiology follow-up	Treatment pathways diverge once MIS-C is suspected	[4, 44, 64]

*RIME* Reactive Infectious Mucocutaneous Eruption, *SJS/TEN* Stevens–Johnson syndrome/toxic epidermal necrolysis, *EM* erythema multiforme, *KD* Kawasaki disease, *IVIG* intravenous immunoglobulin

## Imaging and Histopathology

Imaging and histopathology should be considered supportive rather than core diagnostic or pathognomonic tests. Imaging is most useful for complications or alternative diagnoses, and biopsy helps exclude SJS/TEN, autoimmune blistering disease, infection, or vasculitis when the clinical picture is atypical [4, 5, 9, 51–53].

Ιmaging is not routinely indicated in most children with RIME but may be helpful in select cases with concern for deeper tissue involvement, abscess formation, significant soft-tissue edema or complications involving the airway, orbit, or genitourinary tract. Ultrasound may show inflammatory swelling, increased echogenicity, or fluid collections, while MRI is helpful to better delineate mucosal inflammation, soft-tissue edema and deeper extension when the clinical picture is atypical or severe.

Ηistopathology remains more directly informative. In pediatric RIME, biopsies commonly show epidermal necrosis with a denser inflammatory infiltrate than is typical of SJS/TEN, including lymphocytes, neutrophils, and occasionally eosinophils. Subepidermal blistering may be present in more severe lesions [5–9, 51–53]. Endothelial swelling and focal vasculitic change can also be seen, consistent with the broader picture of cytokine-driven endothelial activation and mucosal injury [51, 52].

Immunohistochemistry may further demonstrate activated T cells, macrophages, and increased expression of inflammatory mediators such as IL-6, TNF-α, and IFN-γ [51, 52]. These findings are not diagnostic on their own, but they support the view of RIME as an inflammatory, parainfectious syndrome and can be particularly useful when differentiating it from SJS/TEN, erythema multiforme, or other inflammatory blistering conditions [5–11, 51–53].

## Long-Term Outcomes and Complications

The prognosis of pediatric RIME is generally favorable, and many children recover fully with supportive care and resolution of the inciting infection. Even so, the short-term burden can be substantial, and long-term sequelae are not negligible, especially after severe ocular or genital disease.

Ocular complications are among the most important. Persistent conjunctival inflammation can lead to adhesions, symblepharon, keratitis, dry eye, and, in severe cases, lasting visual impairment [45, 47, 50, 131]. Oral and genital disease may also heal with scarring, chronic discomfort, or functional limitation [45, 47, 50, 131]. Secondary infections and prolonged pain may complicate recovery in children with extensive mucosal breakdown [45, 47, 50, 131]. Children with significant ocular involvement should have documented ophthalmology follow-up after discharge, because dry eye, keratitis, conjunctival scarring, and symblepharon may appear or persist after the acute mucositis improves [45, 47, 50, 131].

The psychosocial impact should not be overlooked. Hospitalization, inability to eat or speak comfortably, genital pain, and the frightening appearance of mucosal lesions can be distressing for both patients and families. Some children may go on to experience anxiety, social withdrawal, or post-traumatic stress symptoms after a severe episode [4, 44, 45, 47, 50]. For this reason, follow-up should be multidisciplinary when needed and should not end at visible mucosal healing. Pediatric, dermatologic, ophthalmologic, nutritional, and psychological support can all be important parts of recovery in more severe cases.

## Conclusions

RIME in children is best understood as a mucosa-predominant, pathogen-triggered inflammatory syndrome in which host response plays a central role. Across a wide range of infectious triggers, common immunologic themes recur: epithelial sensing of microbial danger signals, activation of innate pathways such as NF-κB, interferon, and inflammasome signaling, recruitment of neutrophils and activated lymphocytes, and amplification through IL-1, IL-6, TNF, and interferon-associated mediators. These pathways help explain why children often present with severe oral, ocular, or genital disease despite relatively limited skin involvement. However, specific immune pathways should be interpreted according to the evidence hierarchy: some are directly supported by RIME/MIRM data, whereas others remain extrapolated from related mucocutaneous or infectious inflammatory disorders [1, 2, 29, 34–37].

At the same time, RIME is biologically heterogeneous. Pathogen-specific mechanisms, host cytokine balance, and likely genetic susceptibility all shape the final phenotype. Emerging proteomic data strengthen this view by identifying recurring inflammatory modules and clinically meaningful endotypes that may eventually support more precise risk stratification and more targeted immunomodulation. Current molecular data support a host-response-centered model but do not yet establish validated cytokine, proteomic, or genetic endotypes for routine clinical decision-making.

For clinicians, the most immediate priorities remain early recognition, exclusion of key mimics such as SJS/TEN, careful search for the infectious trigger, and aggressive supportive care with multidisciplinary involvement when ocular, nutritional, or genitourinary complications are present. For researchers, the next step is clear: longitudinal studies integrating pathogen detection, cytokine profiling, proteomics, and host genetics are needed to define which children are at highest risk of severe or recurrent disease and which therapeutic strategies are most likely to alter the course of illness. Key research needs include prospective pediatric cohorts, standardized microbiological testing, paired acute and convalescent blood and mucosal samples, mucosal immunophenotyping, proteomics with appropriate comparator groups, careful treatment-response documentation, and long-term follow-up of recurrent or ocularly severe cases. 

## Data Availability

No datasets were generated or analysed during the current study.
